# A novel method for precise endoscopic sampling of duodenal microbiota

**DOI:** 10.3389/fcimb.2025.1517751

**Published:** 2025-07-18

**Authors:** Taotao Wei, Gaozhong Dai, Tianye Liu, Yaozhou Tian

**Affiliations:** ^1^ Wuxi hospital Affiliated to Nanjing University of Chinese Medicine, Wuxi, China; ^2^ Affiliated Hospital of Integrated Traditional Chinese and Western Medicine, Nanjing University of Chinese Medicine, Nanjing, China

**Keywords:** endoscopic, duodenal, microbiota, channel plug, 16S rRNA

## Abstract

**Background:**

Previous studies have established a relationship between duodenal mucosa-associated microbiota and overall health. However, sampling duodenal microbiota is technically challenging. Mucosal biopsies collected via endoscopy are the most common approach, but this method risks contamination of the working channel with gastrointestinal contents or extraneous microorganisms.

**Methods:**

This study designed a novel accessory, an endoscopic channel plug, to improve the sampling process by ensuring a clean and sterile working channel, thereby providing more accurate microbiota results.

**Results and conclusion:**

Microbiome analysis of samples collected from the oral cavity, traditional duodenal sampling, and the modified method with the channel plug revealed that samples obtained with the plug exhibited higher PCR product concentrations and a greater number of operational taxonomic units (335). Additionally, 16S rRNA sequencing showed significant differences in the taxonomic composition at both the phylum and genus levels among the different sampling methods. Notably, the novel method group (using the channel plug) contained a higher abundance of *Veillonella*, whereas this genus was less abundant in oral cavity and traditional duodenal samples. Furthermore, the abundance of specific bacterial strains varied significantly between sampling methods. These findings suggest that the use of the channel plug enables more comprehensive microbiota sampling, providing data to support clinical diagnosis of gastrointestinal diseases.

## 1 Introduction

There is a complex and dynamic microbial community in the human gastrointestinal tract, which is closely related to the host’s health and plays a key role in the occurrence and development of many diseases. Therefore, it is essential to analyze the relationship between intestinal flora changes and disease occurrence, progression, and prognosis (Weersma et al., 2020; Xu et al., 2024). In the past, the analysis of the intestinal microbiome was mainly based on the isolation and cultivation of the microbiome, but the culture conditions of anaerobic bacteria in the intestine are relatively harsh and difficult to succeed, which seriously affects the accuracy of the analysis (Jones et al., 2018; Tang et al., 2020). In recent years, sequencing technology for intestinal microbial analysis has developed rapidly, providing great help for clinical gastrointestinal diagnosis and treatment (Damhorst et al., 2021; Athanasopoulou et al., 2023). However, collecting appropriate samples is also crucial for clinical testing. At present, research usually uses endoscopy for mucosal biopsy or suction to take samples, but during the operation, the working channel of the endoscope is inevitably mixed with the contents of the digestive tract or other parts of the bacteria, which will lead to inaccurate sampling (Liang et al., 2020; Xu et al., 2024).

The duodenal mucosa provides crucial information on the gut microbiota, inflammation, infections, and other pathological changes, which can help diagnose and detect a variety of gastrointestinal diseases, so obtaining accurate samples is very important for clinical treatment decisions. A study by Nardelli, Darra, and others has identified significant differences in the composition and diversity of the duodenal mucosal microbiota in patients with metabolic diseases such as fatty liver and diabetes, compared to healthy individuals (Nardelli et al., 2020; Weersma et al., 2020; Kuziel and Rakoff-Nahoum, 2022; Darra et al., 2023). To improve this situation, Shanahan designed Brisbane Aseptic Biopsy Device forceps that can perform targeted biopsy under sterile conditions. However, shortcomings such as the small amount of sample tissue obtained and host DNA interference limit further application (Shanahan et al., 2016). Mottawea introduced a method of flushing the endoscope’s working channel with sterile water, which is simple to implement, but the impact of residual microbial contamination remains unclear (Mottawea et al., 2019). However, swallowable sampling capsules or 3D-printed tablets cannot be widely used in clinical research due to their complex design, high cost, and inability to provide targeted sampling (Tang et al., 2020).

Based on the above situation, a new type of endoscopic channel plug was designed to improve the endoscopic sampling process and maintain the sterility of the working channel to obtain more accurate duodenal mucosal flora samples. The aim is to enhance the accuracy of microbiota analysis, providing a more reliable foundation for clinical research on gut microbiomes.

## 2 Materials and methods

### 2.1 Channel plug accessories and sampling brushes

**Figure 1:**
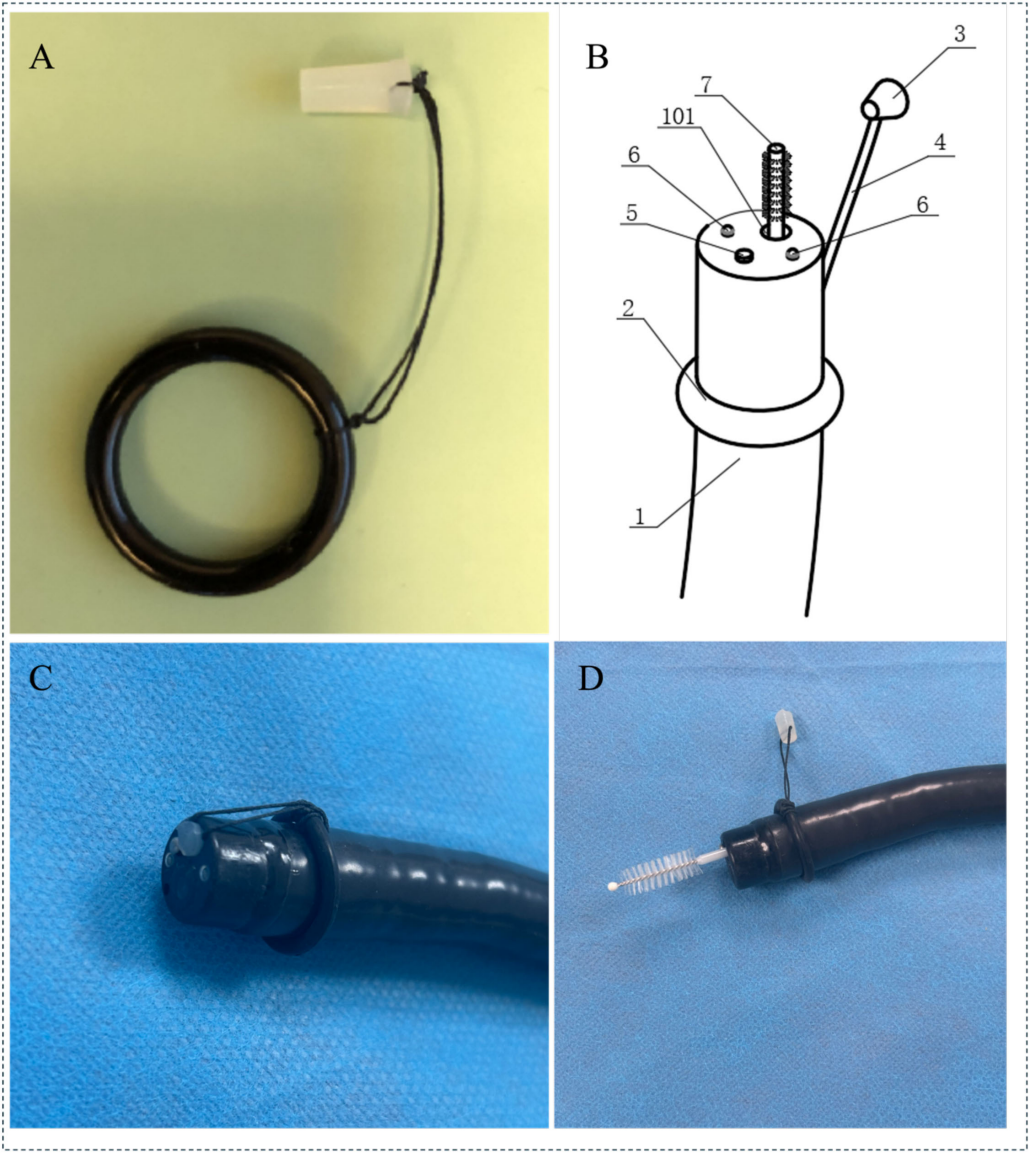
Endoscopic channel plug and sampling brush: **(A)** Components of the endoscopic channel plug; **(B)** Schematic Representation of the Endoscope Structure; (1. Endoscope; 2. Silicone Ring; 3. Silicone Plug; 4. Connector Cable; 5. Objective Lens; 6.LED Light Source; 7. Disposable Sampling Brush; 101.Working Channel); **(C)** The plug fixed to the distal tip of the endoscope; **(D)** Sampling with a cytology brush passed through the endoscopic channel. (The sampling procedure: 1.Sterilize the endoscope and accessories using ethylene oxide; 2.Fix the accessories to the endoscope, insert the scope into the descending part of the duodenum without any suction; 3.Use the sampling brush to push open the silicone plug and brush the mucosal surface; 4.Withdraw the brush, cut off the brush head, and store it in a sterile cryotube; 5.Remove the endoscope and accessories as a whole.).

As shown in **Figure 1**, the accessory is installed at the distal end of the endoscope and consists of a flexible silicone plug connected to a silicone ring. The silicone plug can effectively seal the working forceps channel and maintain the sterility of the working forceps channel during the endoscope insertion process. The silicone ring is fixed to the endoscope body, and its function is that after the sampling is completed, the entire accessory can be removed with the endoscope. The study used a disposable endoscope cleaning brush to brush the bacterial flora sample on the surface of the duodenal mucosa.

### 2.2 Equipment sterilization

In this study, the Pentax EC38-iL gastroscope was used and all endoscopes, accessories, and the silicone endoscopic channel plug were sterilized using ethylene oxide. The sterilization parameters were set as follows: relative humidity at 50%, and ethylene oxide concentration at 600 mg/L, for 5 hours. This was followed by 12 hours of aeration to ensure no residual ethylene oxide gas. The transport, assembly, and sampling procedures for the endoscope were carried out by medical staff, with strict adherence to aseptic techniques.

### 2.3 Patient recruitment and sample extraction

This study was approved by the Ethics Committee of Wuxi Hospital of Traditional Chinese Medicine [SWJW2022062701]. This study has been registered and filed online at the China Clinical Trial Registry [ChiCTR2400089346], and written informed consent was obtained. Fifteen patients were recruited from the Department of Gastroenterology outpatient clinic for physical examination. Exclusion criteria included the use of antibiotics or probiotics. A total of 15 patients participated in this study. Specific information on the patients is shown in **Table 1**.

**Table 1:** Information about the patients in this study.

ID	OR Group	TM group	NM group	Gender	Age
1	1	1	1	Male	31
2	1	1	1	Male	60
3	1	–	1	Female	46
4	1	1	1	Male	28
5	1	–	1	Male	54
6	1	1	1	Male	26
7	1	1	1	Male	45
8	1	1	1	Female	47
9	1	1	1	Female	56
10	1	1	1	Female	30
11	1	1	1	Male	39
12	1	1	1	Female	42
13	1	1	1	Female	32
14	1	1	1	Female	35
15	1	1	1	Male	50

First, oral samples were collected, and oral mucosal surface samples were obtained from the tongue, palate, and pharynx (Oral group, OR group). After positioning the patient, the anesthesiologist performed propofol intravenous general anesthesia and performed a painless endoscopic operation. Before inserting the endoscope, the silicone plug was installed at the front end of the endoscope, and the suction tube connected to the endoscope was disconnected to ensure the sterility of the working forceps channel. During the operation, the endoscopist directly inserted the endoscope into the descending part of the duodenum (D2 area) and then used a disposable cleaning brush to push open the silicone plug and brush the four walls of the mucosa to collect mucosal surface samples from the D2 area of ​​the duodenum (Novel Method, NM group). After the sampling was completed, the cleaning brush was removed, the brush head was cut off, and it was immediately stored in a sterile cryotube. Subsequently, the endoscope was replaced and re-entered into the duodenum D2 region, the forceps channel was flushed with 25 ml of sterile water, and then a disposable cleaning brush was used for sampling (Traditional Method, TM group). All collected samples were incubated at room temperature for 30 minutes and then stored in a -80°C freezer for subsequent analysis.

### 2.4 16S rRNA extraction and amplification

The extraction and amplification of 16S rRNA were performed according to the previous method (Barlow et al., 2021). Fecal DNA was extracted according to the manufacturer’s instructions of FastDNA Spin Kit for Feces (MP Biomedicals, USA) and purified using the AxyPrep DNA Gel Extraction Kit (Axygen Biosciences, Union City, California, USA). Paired-end sequencing was performed on the Illumina MiSeq PE300 platform of Shanghai Majorbio Bio-Pharm Technology Co. Ltd.

### 2.5 Statistical analysis

Data processing and drawing were completed using GraphPad Prism 8.0 software. Experimental data were expressed as “mean ± standard deviation” or “median-interquartile range”. The Kolmogorov–Smirnov method was used to test the normality of the data. For data that conformed to the normal distribution, one-way analysis of variance (ANOVA) and Fisher’s LSD *post hoc* test were used for difference analysis; for data that were not normally distributed, the Kruskal-Wallis nonparametric test and Dunnett’s *post hoc* test were used for difference analysis. P < 0.05 was considered to be significantly different. For high-dimensional data, the FDR (Benjamini-Hochberg method) was used to correct the P value. The Spearman correlation coefficient was used to analyze the correlation between different types of data.

## 3 Results

### 3.1 The impact of different sampling methods on sample quantity

**Figure 2:**
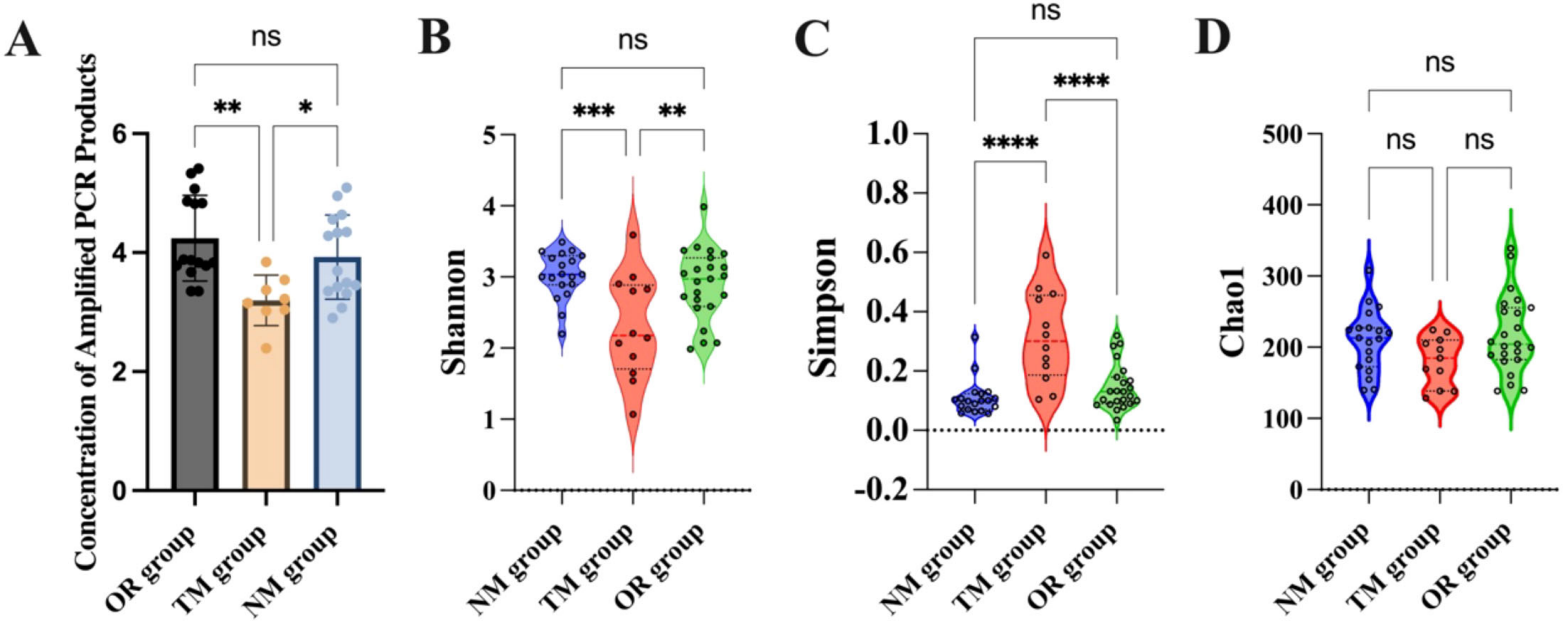
**(A)** Concentration of amplified PCR products; **(B)** Shannon diversity index; **(C)** Simpson diversity index; **(D)** Chao1 diversity estimator. *p < 0.05, **p < 0.01, ***p <0.001, and ****p < 0.0001, and “ns” indicates no statistically significant difference, as determined by one-way ANOVA.

An analysis of the PCR product concentration from samples collected using different sampling methods revealed that the product concentration obtained through the novel method developed in this study was significantly higher than that obtained through the traditional method (**Figure 2A**). This indicates that the endoscopic channel plug can dramatically increase the concentration of the collected samples. Additionally, the microbial alpha diversity from different sampling methods was assessed in **Figures 2B–D**. The Shannon and Simpson indices are commonly used to indicate species diversity within a community, while the Chao1 index reflects species richness. Results showed that the Shannon and Simpson indices were significantly higher in samples collected using the novel sampling method, indicating a notable increase in species diversity. However, the Chao1 index showed no significant difference, suggesting that while the endoscopic channel plug enhances microbial diversity, it does not increase the total species. Tang et al. demonstrated that innovative sampling devices with contamination prevention enhance sample concentration and quality (Tang et al., 2020). Kim et al. reported that fluid aspiration during colonoscopy significantly increased the Shannon and Simpson indices but did not affect Chao1 abundance, which is consistent with our observation that endoscope channel plug attachment increased microbial diversity, but not abundance (Kim et al., 2021).

### 3.2 The impact of different sampling methods on microbiota

**Figure 3:**
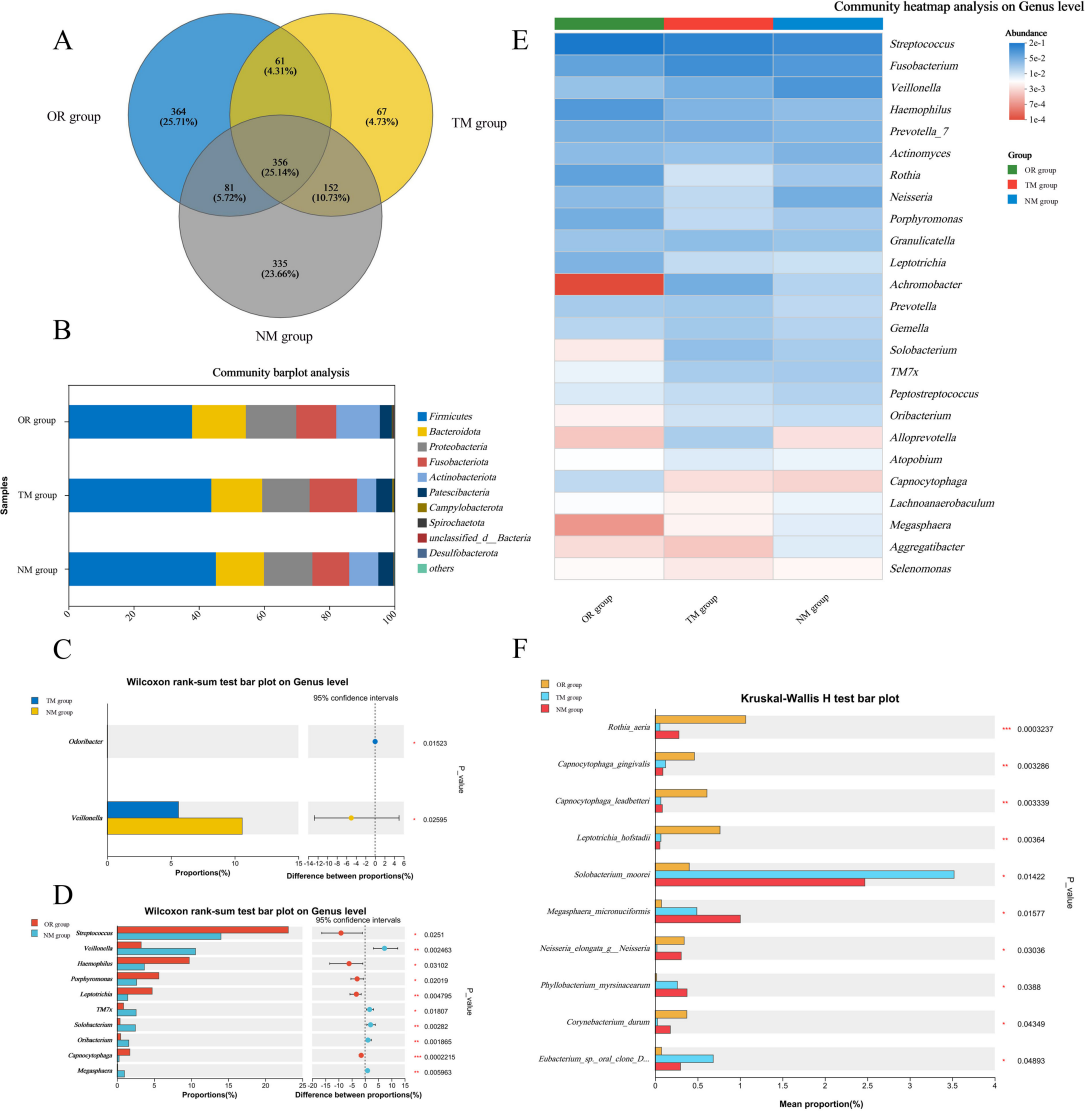
Profiles of mucosal microbiota obtained via different sampling methods. **(A)** Number of operational taxonomic units (OTUs); **(B)** Variations in taxonomic composition at the phylum level across different sampling methods; **(C)** Differences in genus-level composition between the TM group and the NM group; **(D)** Genus-level differences between the oral group and the NM group; **(E, F)** Microbial composition disparities across different sampling methods. *p < 0.05, **p < 0.01, ***p < 0.001, as determined by one-way ANOVA.

**Figure 4:**
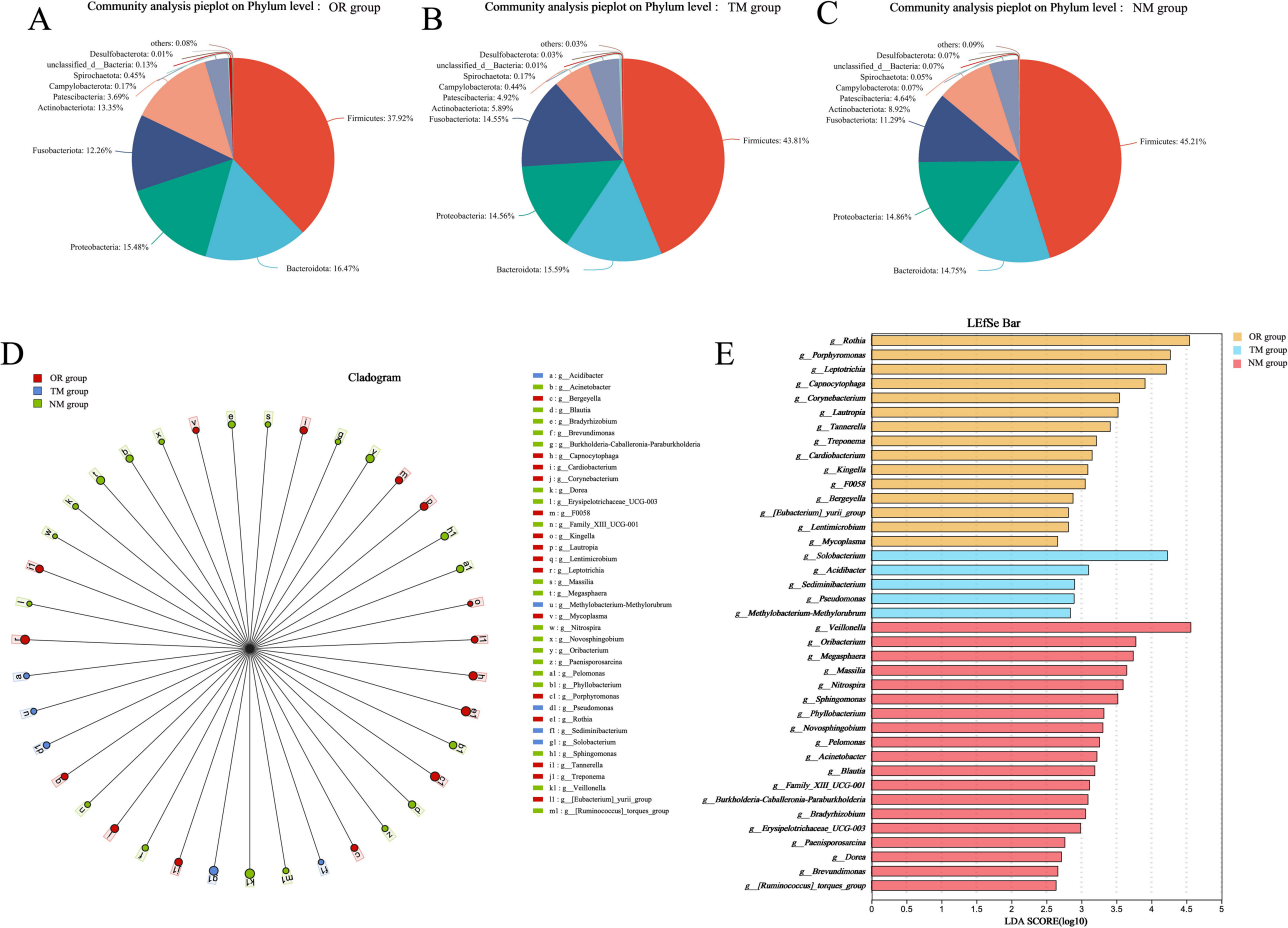
Microbial taxa and distribution according to different sampling methods. **(A–C)** Distribution plots of microbial species across different sampling methods; **(D, E)** LEfSe distribution plot and distribution table.

As shown in **Figure 3A** and **Figures 4A–C**, the total OTU count in different groups revealed that the OTU value in the NM group was 335, while in the TM group, it was 67, indicating that using the channel plug significantly increased the OTU content in the samples. Further analysis of the gut microbiota composition at the phylum level, comparing the endoscopic channel plug sampling group with the ordinary sampling and oral sampling groups, is presented in **Figure 3B**. The results show that the gut microbiota is primarily composed of *Firmicutes*, *Bacteroidota*, *Actinobacteria*, *Proteobacteria*, *Fusobacteriota*, and *Patescibacteria*. The proportion of *Firmicutes* in the NM group was significantly higher than in other groups. *Firmicutes* play a critical role in maintaining gut health and supporting the proper function of the immune system. As well as promoting gut health, they assist in regulating immune responses and protect the gut from infection by maintaining intestinal balance. In contrast, the *Bacteroidota* in the NM group was markedly lower than other groups, and the abundance of *Proteobacteria* was no significant difference with others. These findings demonstrate that the use of the channel plug not only increases OTU abundance but also alters the composition of gut microbial phyla, providing a more accurate reflection of the patients’ gut composition. For instance, the relative abundance of *Fusobacteriota* in the TM group was significantly higher than NM group. An increased abundance of *Fusobacteriota* is associated with *Helicobacter pylori*-induced gastric infections, making it a potential marker for evaluating *Helicobacter pylori* infection. Therefore, these results provide a valuable reference for the clinical application of gastroscopy.

Further analysis at the genus level revealed that the NM group had the higher abundance of *Veillonella*, which plays an important role in infections of the oropharynx and gastrointestinal tract. Penicillin is often used to treat infections caused by this pathogen in clinical. Therefore, using the channel plug can enhance the accuracy and comprehensiveness of clinical sampling. In comparison to the NM group, oral sampling showed significantly higher abundance at the genus level. However, the content of *Veillonella* was notably lower in the oral samples compared to the duodenal samples from the NM group, which may be attributed to differences in sample distribution.

Additionally, LefSE analysis indicated that microbial composition at the genus level varied significantly between different sampling methods. As shown in the **Figure 4**, the NM group had a higher abundance of genera such as *Veillonella*, *Oribacterium*, *Megasphaera*, *Massilia*, and *Nitrospira*. In contrast, the TM group exhibited more *Solobacterium*, *Acidibacter*, *Edimibacterium*, *Pseudomonas*, and *Methylobacterium-Methylorubrum*, while the oral samples contained higher levels of *Rothia*, *Porphyromonas*, *Leptotrichia*, *Capnocytophaga*, *Corynebacterium*, and *Lautropia*. These findings suggest that different sampling methods result in distinct microbial communities.

Compared to the TM group, the microbial diversity of duodenal mucosa in the NM group was significantly increased. Although the genus-level abundance in the NM group was similar to that of the oral samples, the species composition differed considerably. This indicates that sampling using the endoscopic channel plug provides a more objective and comprehensive representation of the duodenal mucosal microbiota, offering technical support for clinical studies.

**Figure 5:**
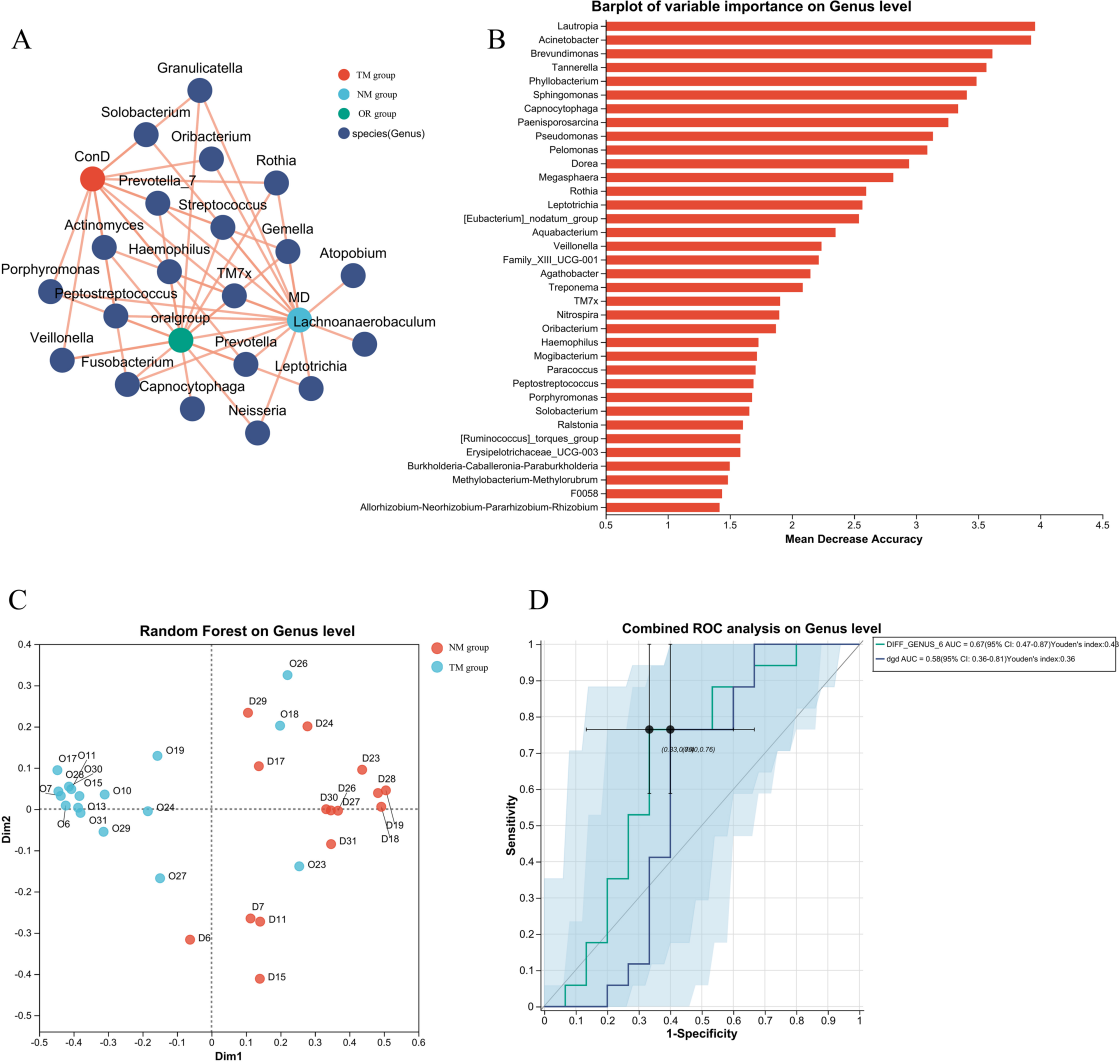
**(A)** Construction of diagnostic models for different groups and network analysis, association analysis, and model prediction; **(B)** Construction of diagnostic models for different groups and Random Forest species distribution statistics in network analysis; **(C)** Construction of diagnostic models for different groups and Random Forest sample distribution and classification in network analysis; **(D)** Construction of diagnostic models for different groups and network analysis combined with ROC analysis results.

**Figure 6:**
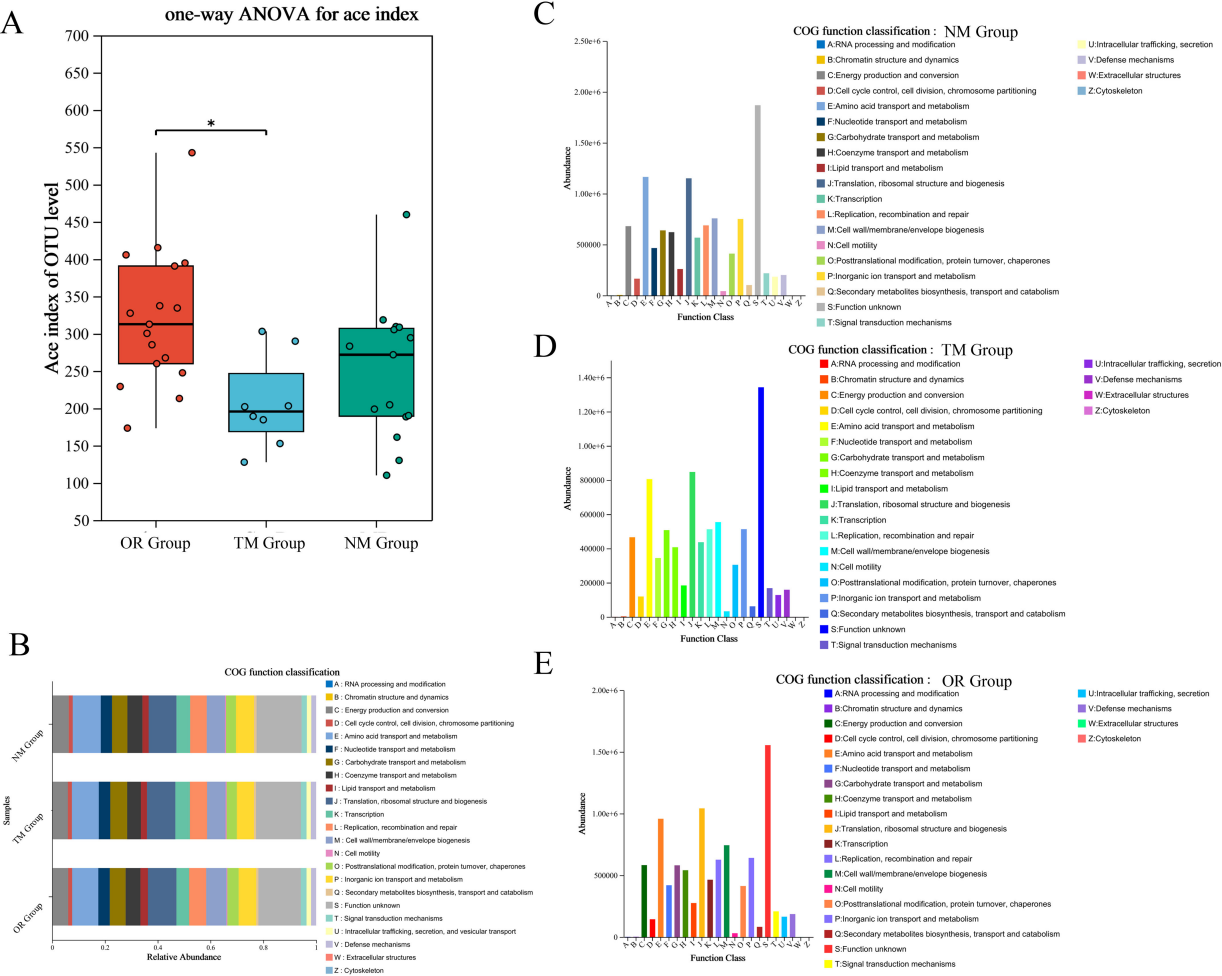
**(A)** Alpha diversity index difference test between different groups; **(B)** Functional prediction using PICRUSt2 across different groups—classification of COG (Clusters of Orthologous Groups) function. **(C–E)** COG function distribution at the species level across different groups.

The construction of models and network analysis, including correlation analysis and model prediction, illustrates the distribution patterns between samples and species (collinearity network). Additionally, it facilitates the study of correlations among species (univariate correlation network) as well as between species and clinical factors (bivariate correlation network). By leveraging network properties, key species involved in the pathogenesis and progression of disease can be identified. As shown in **Figures 5A, B**, there is a clear distinction between the ConD group and the Oral Group. Notably, microorganisms such as Veillonella, Lautropia, and Acinetobacter exhibit strong correlations with the Oral Group. Further Random Forest analysis comparing the MD group and the Oral Group revealed substantial differences between the two groups (**Figure 5C**). Moreover, the combined ROC analysis yielded an area under the curve (AUC) values of 0.67 and 0.58 (**Figure 5D**), indicating a moderate level of accuracy and potential diagnostic value. A comparative analysis of the ACE index across different groups was conducted to infer potential changes in species richness caused by other environmental factors (**Figure 6A**). The results showed that the ACE index of the Oral Group was significantly higher than that of the ConD Group, indicating that the novel sampling technique not only enables a more comprehensive acquisition of sample indices but also achieves highly effective sampling of other unknown components in the sampling environment. Furthermore, PICRUSt2 functional prediction and Clusters of Orthologous Groups (COG) analysis were performed on samples obtained through different sampling methods. A detailed analysis of the COG distributions within each sampling method revealed that, overall, there were no substantial differences among the groups (**Figures 6B–E**).

## 4 Discussion

Gastrointestinal dysbiosis is a characteristic feature of non-communicable diseases such as obesity, cardiovascular diseases, and metabolic disorders. Numerous studies have shown that alterations in microbial diversity and microbial translocation can influence disease progression and may act as independent risk factors in some cases. For instance, the accumulation of *Klebsiella* and *Enterobact*er in oral inflammation can migrate to the gut, causing direct inflammation and indirectly exacerbating intestinal inflammation by inducing pathogenic Th17 cells capable of migrating to the gut (Borriello, 2018; George and Finegold, 2018; Xu et al., 2022). As the initial segment of the small intestine, the duodenum has a specialized mucosal layer that serves as a defensive barrier, protecting against microbial translocation to host cells (Zhou et al., 2020). However, unlike the virulence and toxicity of resident microbiota, translocated microbes in the complex chemical environment of the gut may disrupt the epithelial-gut barrier integrity and mediate pathological and inflammatory processes (Sundin et al., 2020). For example, in functional dyspepsia (FD) patients, duodenal mucosal permeability is correlated with the severity of inflammation. Studies have confirmed changes in the microbial composition, such as a reduction in the abundance of resident mucosal bacteria like *Neisseria* and *Porphyromonas*, alongside the detection of translocated oral bacteria like *Streptococcus salivarius* (Nojkov et al., 2020; van Baar et al., 2020; Rostami et al., 2022).

Due to the relative ease of fecal sample collection, both clinical practice and scientific research often rely on fecal analysis to assess information from the distal gastrointestinal tract. In research, while the distal gut microbiome is often utilized to assess study outcomes, the duodenum is pivotal in digestion and absorption, serving as a crucial link between the stomach and the digestive tract, with its microbiota closely linked to liver diseases (Dreskin et al., 2021). Exploring the influence of duodenal microbiota on host physiology and disease mechanisms is challenging due to the strict growth conditions of microbiota in the proximal small intestine. Different from traditional microbial culture methods, 16S rRNA sequencing and metagenomics are currently the main technologies for studying the gut microbiota (Wauters et al., 2022). In addition, studies of the duodenal mucosa-associated microbiota (MAM) often employ endoscopic biopsy techniques, which, while effective in capturing deeper mucosal bacteria, are not immune to cross-contamination during sample collection (Kim et al., 2024). Therefore, determining the best way to obtain accurate duodenal microbiota samples is critical to advancing clinical diagnosis.

In this study, a specialized endoscopic channel plug was designed to be capable of maintaining sterility within the endoscopic channel, thereby eliminating contamination by non-resident microbiota during sampling. The oral microbiota was used as a control group to compare with the microbial communities obtained via the endoscopic channel plug, in order to assess similarities and differences between them. Because the oral cavity is directly connected to the gastrointestinal tract, serving as the upstream source of microbes that can migrate into the esophagus, stomach, and even the small intestine. Moreover, during endoscopic procedures, the insertion of the scope passes through the oropharyngeal region, potentially introducing oral microorganisms into gastrointestinal samples. Therefore, using oral samples as the control microbiome can evaluate whether there is upstream contamination or microbial reflux. In summary, using the oral microbiota as a control not only enables evaluation of the channel plug’s ability to block non-target microbes but also helps identify possible contamination introduced during the sampling process. By attaching this device to the gastroscope, we were able to perform a detailed analysis of the duodenal microbiota. Our findings showed that the primary taxa in the duodenum were *Streptococcus*, *Veillonella*, *Fusobacterium*, and *Neisseria*, while the oral microbiota, in comparison, contained taxa such as *Rothia*, *Porphyromonas*, and *Leptotrichia* at the genus level, indicating significant differences between the two sites. The use of channel plugs provides more reliable results compared to the obvious disadvantages of traditional sampling methods, especially the dilution of digestive tract bacteria after the endoscope channel plug is washed with sterile water, resulting in a reduced concentration of PCR products of extracted microbial DNA. In addition, the microbial composition of the samples obtained through the channel plug was significantly different from those collected by traditional methods. In the NM group, we observed a higher abundance of genera such as *Veillonella*, *Oribacterium*, *Megasphaera*, *Massilia*, and *Nitrospira*, whereas the TM group exhibited a greater presence of *Rothia*, *Porphyromonas*, and *Leptotrichia*. These findings further highlight the significant differences between sampling techniques and highlight the need to use channel plugs to ensure accurate sampling of duodenal microbiota (Kashiwagi et al., 2020) However, This study is limited by the relatively small sample size (n = 15), which may affect the statistical power and generalizability of the findings. Although the results provide preliminary support for the feasibility and reliability of the proposed sampling method, further validation in larger and more diverse cohorts is warranted to confirm its broader applicability in clinical settings.

Compared to previous methods, the microbiome profile produced by the improved sampling method using endoscopic channel plugs more closely resembles the true microbial composition of the duodenum. This method has advantages in terms of lower cost and simplicity of operation compared to 3D-printed capsules or Brisbane Aseptic Biopsy Device biopsy forceps, making it ideal for a wide range of clinical research applications (Tang et al., 2020; Kim et al., 2021). Similarly, this approach could be extended to the study of the terminal ileum microbiota, facilitating the characterization of the ileum and colon micro communities. In addition to the duodenum, the channel plug device may also be applied to other upper gastrointestinal regions, such as the esophagus and stomach. In the esophagus, it can help prevent contamination during mucosal or microbiota sampling, especially in studies of esophageal dysbiosis. In the stomach, the device can help obtain more representative samples from specific regions while minimizing reflux-related contamination. However, further validation is necessary in these anatomical sites. Notably, by maintaining sterility within the biopsy channel, endoscopic channel stopper enables a wider range of investigations, such as more precise and direct collection of digestive fluid from a specific area during endoscopy, which exceeds the accuracy of traditional pipe-based collection methods. However, this technique is not without limitations. Endoscopic procedures are inherently invasive and require skilled operators, especially in the case of colonoscopy. Further studies are needed to assess whether bowel preparation influences the gut microbiota, and to evaluate the procedural complexity, time consumption, and patient tolerance associated with the use of the endoscopic channel plug. These factors must be carefully considered when applying this method in clinical practice. This version is well-suited for a highly specialized academic audience, emphasizing the scientific precision and clarity necessary for professional writing. Metabolic and functional analyses of microorganisms obtained through different sampling methods revealed no significant differences, indicating that the novel channel plug primarily enhances sample richness without affecting microbial metabolic pathways or functions. This finding is consistent with the expected outcomes of this study.

## 5 Conclusion

Alterations in gastrointestinal microbiota are clinically associated with conditions such as obesity, cardiovascular disease, and metabolic disorders. However, obtaining microbiota from the duodenal mucosa—a key site connecting the stomach and intestines—remains challenging due to frequent contamination by intestinal contents. In this study, we developed a novel channel plug designed explicitly for the proximal section of gastrointestinal endoscopes. This plug effectively maintains sterility within the endoscopic channel, minimizing contamination from non-resident microorganisms during sampling. Comparative analyses of microbiota collected using different sampling methods revealed that mucosal microbiota obtained with the channel plug exhibited higher PCR product concentrations and greater OTU counts. Additionally, the taxonomic composition and abundance differed significantly between sampling methods. The NM group showed higher relative abundances of genera such a*s Megasphaera*, *Massilia*, and *Nitrospira*, whereas the TM group had elevated levels of *Rothia*, *Porphyromonas*, and *Leptotrichia*. These findings suggest that the use of the endoscopic channel plug enables more precise and reliable acquisition of duodenal mucosal microbiota, providing some support for gastrointestinal endoscopic assessments and clinical diagnostics.

## Data Availability

The raw data supporting the conclusions of this article will be made available by the authors, without undue reservation.
